# Adjuvant Gabapentinoids Versus Muscle Relaxants to Aid Opioid Discontinuation in Older Adults

**DOI:** 10.1093/geroni/igaf122.360

**Published:** 2025-12-31

**Authors:** Kevin Pritchard, Chun-Ting Yang, Xiaojuan Liu, James Wilkins, Dae Hyun Kim, Joshua Lin

**Affiliations:** Brigham and Women’s Hospital, Boston, Massachusetts, United States; Brigham and Women’s Hospital, Boston, Massachusetts, United States; Brigham and Women’s Hospital, Boston, Massachusetts, United States; McLean Hospital, Belmont, Massachusetts, United States; Hebrew SeniorLife, Boston, Massachusetts, United States; Brigham and Women’s Hospital, Boston, Massachusetts, United States

## Abstract

We compared the likelihood to discontinue chronic opioid therapy after initiating gabapentinoids vs. muscle relaxants among community-dwelling older adults with chronic back pain. We conducted a retrospective cohort study of adults ≥65 years of age in U.S. fee-for-service Medicare data (2013-2018) and Optum Clinformatics® Data Mart Database (2004-2023). We included community-dwelling individuals diagnosed with chronic back pain and opioid use lasting >90 consecutive days, who newly initiated gabapentinoids or muscle relaxants. Our primary outcome of opioid discontinuation was defined by an absence of a refill within 60 days of the supply ending. Secondary outcomes included all-cause mortality and all-cause hospitalization. We controlled for 95 covariates using propensity score 1:1 matching. During the 1-year follow-up, we used Cox proportional hazard regression to estimate hazard ratios (HR) and generalized linear models to estimate risk difference (RD). We pooled database-specific estimates with inverse-variance weighting. We matched 17,063 muscle relaxant and gabapentinoid initiators from Medicare (Mean age [SD]=74 [6] years, female=67%, White=74%) and 138,141 from Optum (Mean age [SD]=74 [7] years, female=71%, White=88%). Compared to muscle relaxant initiators, gabapentinoid initiation was not associated with the rate of discontinuing opioids (HR [95% CI], 0.97 [0.84, 1.11]; RD [95% CI] per 1,000 PYs, -55.4 [-191.3, 80.6]). However, gabapentinoid initiation was associated higher rates of death (HR [95% CI], 1.08 [1.01, 1.16]; RD per 1,000 PYs, 1.6 [0.4, 2.9] and hospitalization (HR, 1.09 [1.06, 1.11]; RD per 1,000 PYs, 33.4 [14.5, 52.4]). Compared to muscle relaxants, initiating gabapentinoids was associated with a higher likelihood of adverse events.

